# Artificial Intelligence and/or Machine Learning Algorithms in Microalgae Bioprocesses

**DOI:** 10.3390/bioengineering11111143

**Published:** 2024-11-13

**Authors:** Esra Imamoglu

**Affiliations:** Department of Bioengineering, Faculty of Engineering, Ege University, Izmir 35100, Turkey; esra.imamoglu@ege.edu.tr

**Keywords:** artificial intelligence, machine learning algorithms, support vector machine, genetic algorithm, decision tree, random forest, artificial neural networks, deep learning, internet of things, microalgae

## Abstract

This review examines the increasing application of artificial intelligence (AI) and/or machine learning (ML) in microalgae processes, focusing on their ability to improve production efficiency, yield, and process control. AI/ML technologies are used in various aspects of microalgae processes, such as real-time monitoring, species identification, the optimization of growth conditions, harvesting, and the purification of bioproducts. Commonly employed ML algorithms, including the support vector machine (SVM), genetic algorithm (GA), decision tree (DT), random forest (RF), artificial neural network (ANN), and deep learning (DL), each have unique strengths but also present challenges, such as computational demands, overfitting, and transparency. Despite these hurdles, AI/ML technologies have shown significant improvements in system performance, scalability, and resource efficiency, as well as in cutting costs, minimizing downtime, and reducing environmental impact. However, broader implementations face obstacles, including data availability, model complexity, scalability issues, cybersecurity threats, and regulatory challenges. To address these issues, solutions, such as the use of simulation-based data, modular system designs, and adaptive learning models, have been proposed. This review contributes to the literature by offering a thorough analysis of the practical applications, obstacles, and benefits of AI/ML in microalgae processes, offering critical insights into this fast-evolving field.

## 1. Introduction

Microalgae stand out as a highly promising option because of their remarkable photosynthetic efficiency, sustainability, and eco-friendly characteristics [1]. They exhibit a rapid growth rate and produce biomass at significantly higher levels compared to traditional crops [2,3]. Moreover, microalgae do not compete for arable land or freshwater resources, making them particularly advantageous for mass production in areas where these resources are limited [2,4]. These microorganisms contribute to 50% of the world’s oxygen supply [5,6] and form the base of the aquatic food web, owing to their well-balanced amino acid and lipid compositions [5,7]. Microalgae serve as a sustainable power source, are nontoxic, and hold considerable promise for reducing carbon dioxide [2]. Research indicates that the production of one kilogram of algae biomass captures approximately 1.83 kg of CO_2_ [8,9]. This finding indicates that the cultivation of microalgae biomass could potentially eliminate approximately 40,600 tons of CO_2_ from the atmosphere each year [8].

Algae biomass can be transformed into a variety of valuable products [10], including proteins, pigments, glycerol, biofuels, and biochemicals [11,12,13,14]. The primary components of microalgae biomass are carbohydrates and lipids [11]. Beyond generating biofuels, these biomolecules can be utilized to create various biochemicals, including nutritional supplements, beauty products, pharmaceutical ingredients, fertilizers, and various high-value substances [11,15].

Commercial cultivation often focuses on species, such as *Chlorella* and *Spirulina*, which can have protein levels surpassing 51% of their dry mass, significantly higher than the 30–40% protein found in soybeans [16,17]. Furthermore, antioxidants such as carotenoids are abundant in species, such as *Haematococcus* and *Dunaliella* [18]. Microalgae also contain nearly all essential vitamins. Due to these remarkable characteristics, microalgae are extensively used in various industrial, environmental, and bio-refinery applications [16]. Currently, the commercial cultivation of microalgae primarily focuses on producing valuable compounds, such as nutraceuticals, pharmaceuticals, and food supplements. The market prices for various microalgal-derived components are approximately $0.20 per gram for proteins, $0.41 for lipids, $0.10 for carbohydrates, and $115 for carotenoids [1]. Additionally, the global market for microalgae-based products is projected to grow from $32.60 billion in 2017 to $53.43 billion by 2026 [2,19].

Many studies have investigated the extraction of various bioproducts from microalgae [10]. The microalgae processes involve both upstream processing (USP) and downstream processing (DSP) [10,20]. In the upstream stage, cultivating microalgae presents a complex challenge because of its biological characteristics. Numerous uncontrollable parameters, such as production conditions and reactor configuration, can significantly impact biomass productivity [10]. Particular cultivation parameters, including temperature, inoculum ratio, pH, reactor type, light intensity, CO_2_ concentration, and nutrient levels, differ based on the species of microalgae being utilized. These factors are tailored to meet the specific needs of each microalgae species. Therefore, creating optimal growth conditions tailored to each strain is crucial for maximizing treatment efficiency and biomass yield [10]. Microalgae cultivation is recognized as a significant advancement in biotechnology. Projections indicate that, by 2036, the global market for microalgae-based biomass could produce 5000 tons of dry biomass annually, generating approximately USD 1250 million in revenue [21,22].

The large-scale production of microalgae biomass faces several significant challenges, including the high costs associated with photobioreactors (PBRs), the necessity of a sustainable growth medium, and the time-consuming nature of algal growth monitoring techniques [21]. While outdoor open raceway pond cultivation allows for the use of sunlight and atmospheric CO_2_ to promote microalgae biomass production, environmental conditions can be highly variable, changing both daily and seasonally. This variability complicates the prediction of outdoor open raceway pond productivity, requiring extensive physical measurements and site-specific calibrations [5]. Accurate forecasting of outdoor open raceway pond productivity is crucial for informed decision making, site selection, and cost optimization in commercial operations. Factors such as environmental temperatures, system circulation, and light availability contribute to the difficulty in controlling these systems, often resulting in lower production rates [5]. In contrast, closed PBRs offer more consistent operational conditions and can achieve higher growth rates [23]. Reports indicate that the volumetric productivity of PBRs can be up to 30 times greater than that of open ponds, largely due to the controlled environment and the reduced risk of contamination in PBR systems [8,24].

The downstream process involves extracting and purifying valuable bioproducts from microalgae biomass, utilizing various systems and techniques [10,25]. There is no strict sequence for whether microalgae biomass should be harvested prior to the extraction of bioactive products; these steps can occur simultaneously or in reverse order, depending on the specific production requirements [8]. Harvesting separates the microalgae biomass from the growth medium, which contains residual salt. The harvested biomass was processed in the extraction phase. Several methods can be used to harvest microalgae, including filtration, centrifugation, flocculation, and flotation [8,26]. Traditional harvesting methods, such as centrifugation and chemical flocculation, account for up to 30% of total costs and 50% of total energy consumption, making them less practical for frequent harvests [27].

Drying is a significant energy-demanding step in the downstream processes. Therefore, effective management of the drying process is crucial for reducing costs and making value-added products economically viable [11]. Research indicates that approximately 75% to 85% of the energy used in algal biorefineries is dedicated to drying microalgae [28]. Consequently, lowering operational costs associated with drying through efficient processes is essential for the commercial success of microalgae products [10]. When pretreating microalgae biomass and extracting biomolecules, several key factors must be considered to maximize yield. These include temperature, time, the selection of the solvent, solvent proportions, and the extraction method used. However, many conventional techniques face challenges such as long treatment times and low yields [11,29].

To enhance the yield of the desired bioactive compounds, it is essential to identify the ideal values of the parameters influencing the pretreatment and extraction processes [11]. Current extraction and quantification technologies often fall short in areas such as cost-effectiveness, significant energy use, extended extraction durations, and low yields [30]. Several green and environmentally friendly extraction methods, such as ultrasonic-assisted extraction (UAE), microwave-assisted extraction (MAE), pressurized liquid extraction (PLE), and enzyme-assisted extraction (EAE), have demonstrated varying levels of success [30,31]. Nonetheless, these techniques require further refinement to address issues such as high validation costs, energy demands, extended extraction durations, and suboptimal yields. There is a pressing necessity to lower input costs in the extraction process to make these methods more feasible [30,32].

Recent advancements in microalgae technology have concentrated on reducing production costs, as the current expenses associated with cultivation and harvesting hinder adequate profit margins [2]. To overcome these challenges, researchers initially utilized traditional mathematical modeling and simulation techniques; however, currently, the integration of artificial intelligence (AI) into processes is used to seek solutions to all challenges [30]. By digitalizing microalgae cultivation and harvesting, operational costs can be significantly minimized [11]. AI technology can enhance PBR performance by ensuring consistent and optimal biomass production. This goal is achieved through the use of interconnected sensors that monitor microalgae development, allowing for adjustments in conditions as needed. Machine learning (ML) algorithms can help identify the ideal growth parameters [21]. Implementing Internet of Things (IoT) and AI technologies in microalgae processes holds great promise for advancing sustainability across three key areas: social, economic, and environmental [8].

This review focuses on identifying current trends and methods in AI/ML and exploring their impact on microalgae processes. It also addresses the challenges and advantages of integrating these technologies. The novelty of this review lies in its comprehensive examination of the intersection between AI/ML technologies and microalgae processes, an area that is still emerging. It highlights how AI/ML can be leveraged to optimize microalgae production, offering insights that go beyond traditional methods. This review fills gaps in the literature by systematically analyzing the real-world applications, challenges, and benefits of AI/ML in this specific domain while also addressing the scalability of these processes, which has been underexplored in existing studies. It provides a fresh perspective on how data-driven approaches can improve efficiency, yield, and process control in microalgae biotechnology.

In this study, a comprehensive search was conducted using the Scopus and Web of Science databases, and 117 relevant articles were ultimately selected. The search focused on publications from 2020 onwards, with 80 articles meeting this criterion to ensure the inclusion of the latest advancements. Specific keywords were used—artificial intelligence, machine learning, microalgae, and microalgae processes—to guide selection and ensure relevance to the field. This approach allowed the capture of a broad yet focused range of studies directly related to AI applications in microalgae bioprocesses.

## 2. Transition from Traditional Mathematical Modeling and Simulation to AI/ML in Microalgae Processes

Bioprocess models play a crucial role in examining how various complex factors influence metabolite accumulation, optimizing processes and lowering operational costs [33]. Among the kinetic models used, Monod kinetics is commonly applied to predict the growth of microalgae and the accumulation of metabolites [11,33]. These models are valuable for forecasting experimental outcomes, fine-tuning equipment, and minimizing experimental expenses [34]. However, the data utilized in these modeling studies are static and do not adapt or improve over time. As a result, applying these models on an industrial scale poses challenges regarding the accuracy of their outcomes. The cultivation conditions for microalgae can differ significantly depending on the strain, local weather, cultivation location, and the chemical properties of the nutrients used. These variations can significantly affect the reliability of the model results [8]. In contrast, machine learning models offer greater versatility compared to traditional mathematical approaches, such as those based on the Monod equation, as they do not necessitate prior knowledge of growth dynamics. Additionally, simulations of growth using machine learning are highly adaptable, allowing the integration of other factors that impact growth, such as temperature and nutrient availability. This kind of integration can be challenging for traditional mathematical models, particularly in scenarios involving variable light conditions [27].

Mathematical models can predict solubility to some extent, whereas simulation models provide insights into the mixing efficiency of microalgae processes. These mathematical models form the basis of Computational Fluid Dynamic (CFD) models [34]. The CFD is commonly used to analyze fluid flow, multiphase interactions, heat and mass transfer, and combustion processes [35,36]. However, CFD methods alone are insufficient for understanding the relationships between parameters such as speed, temperature, and agitation. Utilizing AI models on CFD results enables the identification of these variations [34]. In modeling and simulation strategies, the improper choice of machine tools can result in a range of issues, affecting long-term operations and causing immediate functional problems, some of which may be severe and irreparable [11].

To optimize the reactor configuration and increase production efficiently, it is crucial to maintain ideal conditions such as pH, temperature, CO_2_ supply, and dissolved O_2_ levels. To accomplish this, the creation of advanced models and the implementation of complex control procedures are typically required. Traditionally, this has been achieved by evaluating different design configurations using integrated physical models that merge CFDs with kinetic modeling. However, this approach can be computationally challenging and unstable when applied to large-scale systems. Such difficulties lead to extensive computing demands and impractical mathematical optimization. To address these issues, integrating physical models with data-driven deep learning techniques can provide a viable solution [10].

Response Surface Methodology (RSM) is a conventional statistical technique commonly employed for modeling and optimizing the extraction of bioactive substances [30,37]. It effectively reduced the number of experimental runs by optimizing the independent variables and accurately predicting the responses. However, a significant drawback of the RSM is its inability to adequately describe nonlinear regression equations [38,39]. While the RSM is commonly chosen for modeling and optimizing parameters in the pretreatment and extraction of compounds, it has multiple obstacles and constraints. Many optimization studies fail to identify true optimal points, often owing to the inappropriate determination of factorial ranges. This constraint can result in optimum points falling outside the experimental region, leading to the incorrect identification of optimal points, whether for maximum or minimum response values. Consequently, this action can yield uncertain and ineffective optimization methodologies. With advancements in computational techniques and digitalization, artificial neural networks have emerged as an innovative approach for various scientific challenges [11]. As reported by Srivastava et al. [39], the highest optimized condition achieved a 99.16% conversion efficiency of oil to fatty acid methyl ester using a genetic algorithm, whereas the RSM yielded a slightly lower conversion efficiency of 98.01% for *Chlorella* CG12.

**Figure 1:**
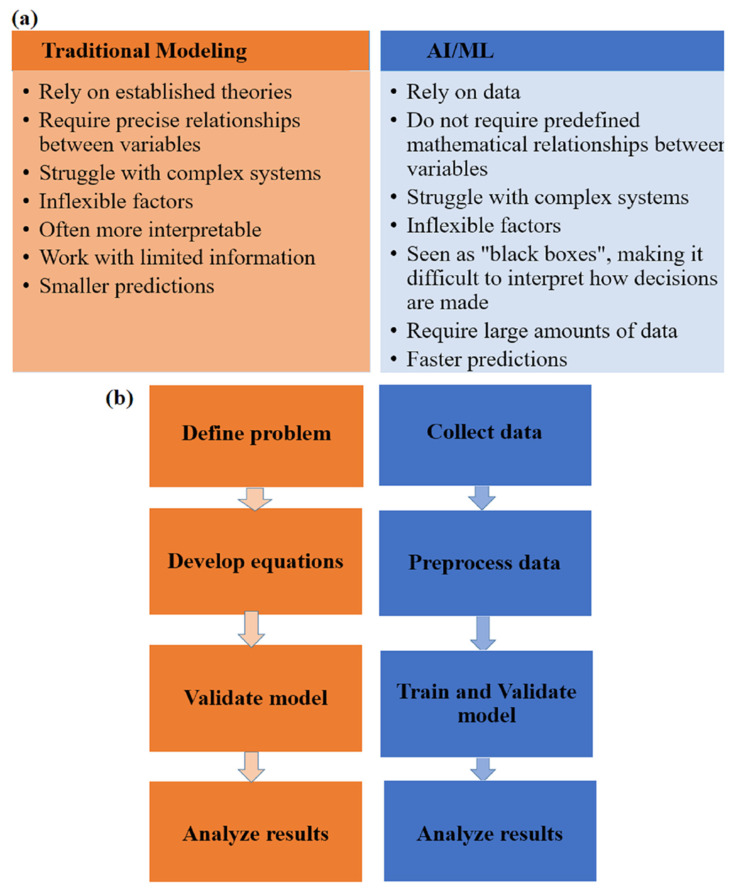
Traditional modeling and AI/ML technique: (**a**) comparison of traditional modeling with AI/ML technique; (**b**) pathways of traditional modeling and AI/ML technique.

As industries began to apply microalgae technology, there was a growing need for real-time monitoring and predictive capabilities that traditional methods struggled to provide. Thus, the transition from traditional mathematical modeling and simulation to AI and ML has started as a gradual process driven by the recognition of the constraints of traditional methods, the accumulation of rich datasets, and advancements in technology. Traditional simulations and modeling are valuable for precise and well-understood scenarios in which detailed mathematical relationships are known. They require complete datasets and are less flexible for handling complex nonlinear interactions (Figure 1a). On the other hand, machine learning tools offer flexibility and adaptability in modeling complex systems with less need for predefined relationships and provide faster predictions than traditional simulations, which can be time consuming, especially for complex systems. These systems demonstrate proficiency in managing incomplete data, handling nonlinearities, and integrating multiple variables (Figure 1b).

## 3. Artificial Intelligence

The term “artificial intelligence” (AI) was first coined by McCarthy in the 1950s, suggesting the idea of creating machines capable of performing tasks usually performed by humans [40,41]. AI encompasses the capability of machines to mimic human intelligence, allowing them to engage in complex activities, such as problem solving, making choices, and identifying objects [41,42]. It is a vital area within computer science and is recognized as one of the key technologies of the twenty-first century [43]. In 2019, the global artificial intelligence market was valued at USD 27.23 billion. It is projected to grow significantly by 2027, reaching an estimated USD 266.92 billion. This increase corresponds to a compound annual growth rate (CAGR) of 33.2% [8]. AI techniques enable the understanding of how various components interact and whether their combinations can outperform traditional physical experiments in terms of speed [33]. By leveraging its rapid problem-solving abilities, AI significantly alleviates the need for human labor, physical materials, and financial investment. This effectiveness is crucial for the advancement and widespread adoption of AI [34].

The application of AI methods to outcome prediction requires several key steps. The first is the need for a large dataset to act as the training set. The model is trained, and its parameters are adjusted to better understand the relationships within the data. When the changes in the parameter values become minimal, this indicates that the training process is complete. After training, the model can be utilized to forecast the outcomes based on different input variables. Unlike traditional mathematical models, AI approaches provide a more intuitive way to represent data variations [34]. Even in the face of data scarcity, AI can infer missing data and adjust models using the limited information available, thereby improving accuracy [44]. On the other hand, AI systems demand significant computational energy for data processing and analysis. As the volume of data increases, the need for computational power increases, necessitating algorithms that can efficiently handle large datasets while minimizing power consumption [45].

The application of AI is becoming increasingly popular in microalgae research, as its algorithms can effectively address the complexities of uncertain biosystems [46]. AI has the potential to significantly boost the productivity of microalgae cultures. By leveraging AI, researchers can optimize cultivation conditions with greater precision, enhancing the accuracy of identifying, classifying, and quantifying various algal strains and their growth patterns [2,21]. This innovation lays the groundwork for developing automated cultivation systems, which could lower the costs of harvesting and extracting bioproducts, thus improving the efficiency and economic viability of microalgae biotechnology [2]. Furthermore, in the context of microalgae-based biorefineries, AI technologies can accelerate the optimization process, provide valuable predictive analytics, and help reveal system dynamics and uncertainties [46].

It can be challenging to draw distinct boundaries between various topics in AI and machine learning (ML). Understanding the differences between these concepts is crucial for a solid understanding of AI. The AI serves as the broadest framework. ML falls under the umbrella of the AI. In the realm of machine learning, neural networks and deep learning are considered subfields, and deep learning acts as a more focused subset of neural networks [47].

### 3.1. Machine Learning

As a field of AI, machine learning (ML) has evolved significantly, both in its theoretical foundations and practical applications, demonstrating considerable success [48,49]. ML is an interdisciplinary field that combines elements from various domains, including statistical methods, data extraction, probability models, information theory, and algorithmic evaluation [49,50]. ML aims to empower machines to address problems independently by processing data gathered from diverse sources, such as temporal datasets and statistical evaluations [10,51]. ML focuses on identifying and generalizing the relationships between inputs and outputs using inductive inference, which enables machines to make informed decisions in previously unseen contexts [10].

The ML process consists of three main phases: training, cross-validation, and testing [10]. In the first stage, the algorithm analyzes a large dataset to extract insights, allowing the model to undergo effective training [8]. By modifying the parameters based on the training data, the ML model can understand the fundamental patterns present in the data [10]. In the cross-validation phase, a separate validation dataset is utilized to refine the hyperparameters of the model, which are also known as tuning parameters. This step helps identify the most effective model by optimizing the hyperparameters (referred to as hyperparameter tuning or optimization). This process aims to discover the best possible solution while minimizing the computational resource usage and time [10,52]. Finally, the chosen optimal model is evaluated in the testing phase, and its performance is assessed using an independent dataset. This step ensures that the designed and optimized model can effectively make predictions [10,53].

ML tools offer sophisticated methods for assessing, forecasting, and managing uncertainties in microalgae processes [11]. These tools hold significant promise for data analysis, allowing for increased flexibility regarding the types of data used for optimization and predictions. The use of ML has demonstrated its capacity to facilitate rapid process optimization. By harnessing extensive datasets and diverse information sources, such as experimental parameters, sensor readings, and images or scans, ML can help uncover crucial relationships among the characteristics of the constructs. However, a key challenge in developing ML models is that training these models on large datasets can be both time-intensive and expensive [54].

In practical applications, ML can be utilized in real-time operations for predictive modeling, which helps to maintain system stability and enhance the efficiency of microalgae processes [55]. By integrating ML into microalgae cultivation, it is possible to forecast and regulate growth, thereby aiding the microalgae industry in achieving accurate predictions of biomass production. Unlike traditional mathematical or statistical modeling, ML is better suited for large-scale industrial applications, allowing for the effective management of numerous uncertainties that may arise during a process. Given the daily generation of substantial amounts of data from industrial operations, the ML model can be seamlessly integrated into the workflow and updated monthly using new data [8]. Recently, ML has found extensive applications in pharmaceutical and biotechnology companies including Amgen California, Bayer, Eli Lilly, Johnson & Johnson, Merck & Co., and Pfizer. These companies utilize ML for various purposes such as discovering new drugs, identifying biomarkers, diagnosing dis eases, and conducting clinical trial research [30,56].

ML algorithms are adept at predicting nonlinear interactions and managing multivariate data derived from microalgae processes. These algorithms can leverage the existing literature to examine the quantitative correlations between input variables and resultant outputs. This approach is significantly more efficient than traditional comparative analyses because it minimizes the time required to evaluate datasets from individual studies [57]. Although ML algorithms are capable of handling missing data, managing multivariate datasets, and predicting nonlinear relationships, it remains essential to choose the most appropriate algorithm for a specific problem [10]. The ML algorithms include supervised, unsupervised, semi-supervised, and reinforcement learning techniques [43].

Supervised learning involves using both input and output data, allowing the algorithm to understand the connection between them. This approach is commonly applied in classification and prediction tasks, making it particularly valuable for optimizing industrial processes and forecasting production [8]. Examples of ML models based on supervised learning include support vector machines and random forests [58]. In contrast, unsupervised learning relies solely on input data. The algorithm makes predictions by analyzing the input data; if these predictions are inaccurate, the algorithm must be refined. With continued exposure to data, the accuracy of the algorithm improves. Unsupervised learning is frequently utilized for clustering tasks [8]. Internet of Things (IoT) systems can incorporate both supervised and unsupervised learning techniques, depending on the specific application. In addition, semi-supervised learning merges the aspects of both supervised and unsupervised learning. Once the training process is complete, the resulting model can categorize, forecast, or group new examples based on the knowledge gained throughout the training. Importantly, the learning process is ongoing; fresh data generated from operations can further enhance the efficiency and accuracy [8]. Decision trees and neural networks are examples of models that can be constructed using semi-supervised learning. Reinforcement learning, on the other hand, focuses on maximizing an incentive signal by exploring various actions within ML models that interact with their environment [43]. This approach can also be employed in developing genetic algorithms.

The most frequently utilized ML algorithms are support vector machines, genetic algorithms, decision trees, and random forest algorithms [10].

#### 3.1.1. Support Vector Machine

**Figure 2:**
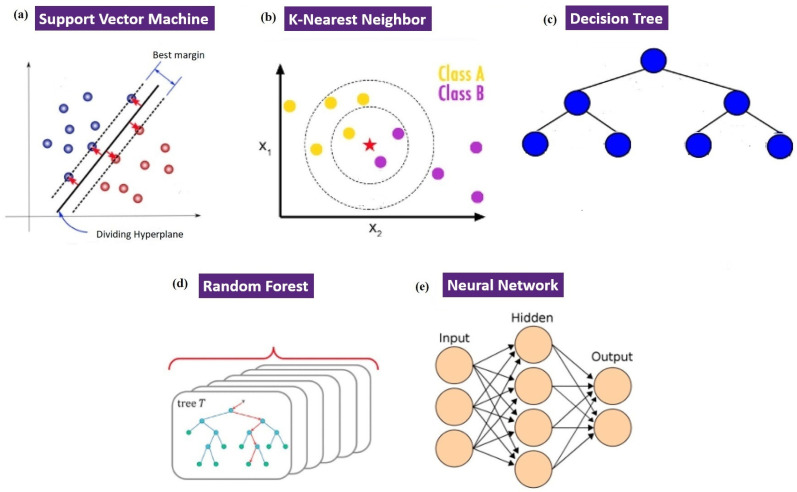
Different AI/ML algorithms [62,63]: (**a**) support vector machine; (**b**) k-nearest neighbor; (**c**) decision tree; (**d**) random forest; and (**e**) neural network.

A Support Vector Machine (SVM) is a robust and flexible algorithm used for supervised learning. It can be used for different functions, such as linear and nonlinear categorization, forecasting, pattern identification, and regression [30,59]. The algorithm works by creating a decision boundary, called a hyperplane (Figure 2a), which divides the data in a multidimensional space into different classes. This action allows the classification of new and unseen data points. Support vectors are crucial for identifying the optimal hyperplane, as they help maximize the margin between the boundary and the closest data points in a multidimensional space [10]. The SVM uses a method called the kernel method to efficiently separate data that are not linearly separable [10]. This method relies on converting the main data into a higher-dimensional space, where clear separation can be achieved. The choice of kernel function is crucial, especially in nonlinear SVMs, as it directly affects the performance of the model [60]. Several kernel functions are frequently utilized, including the linear kernel, polynomial kernel, radial basis function (RBF), and sigmoid function [49,61]. Research has shown that the selection of a kernel function significantly influences the effectiveness of the SVM model. The SVM algorithm has proven effective in multiple areas, such as microalgae classification and wastewater treatment [10]. It considers multiple variables, such as physiological traits and metabolic interactions, to predict the compatibility between different strains and guide the selection of suitable co-cultures [44].

SVMs are particularly suitable when users require more control over aspects such as error tolerance and the choice of the kernel function [57]. Owing to its adaptability in handling sparse training data, it can mitigate the likelihood of overfitting. This step is accomplished by maximizing the distance between the two classes of vectors, while reducing errors within the training data. An SVM is also resilient in dealing with uncertain data and can effectively handle nonlinear relationships between input and output variables [64]. However, one of the primary challenges with SVMs is tuning the hyperparameters, particularly selecting the optimal kernel function and regularization parameter (Table 1). Another limitation is the long computational time required to work with large datasets. Poor data collection and inadequate pre-processing can also negatively impact model performance [30,64]. Additionally, the SVM tends to have inefficient training performance when managing large datasets and is sensitive to absent values. Consequently, when using an SVM, it is essential to carefully consider aspects such as the choice of kernel function, the size of the dataset, and how absent data are handled [10].

#### 3.1.2. Genetic Algorithm

A genetic algorithm (GA) is an optimization technique derived from the process of natural selection and genetic evolution, involving mechanisms such as selection, crossover, and mutation [10,53]. In the GA, the input data form an initial population, which represents a set of possible solutions. This population can be generated randomly or using heuristic knowledge. The less-fit individuals in the population are eliminated, and, to explore new areas of the solution space, variations are introduced in the new population through recombination operators such as crossover and mutation. Over successive generations, the fittest individuals persist, ultimately leading to the most optimized solution [39].

A key benefit of using this model is that it seeks an optimal solution by avoiding local minima. Studies have shown that GAs outperform other approaches in solving combinatorial optimization problems [10]. Additionally, GAs are versatile because they can work with both continuous and discrete variables, enabling a broader search of the parameter space to identify optimal conditions [30]. They have been effectively applied to optimize parameters for microalgae growth and resource recovery [10]. However, a major drawback of GAs is the risk of premature convergence, which can occur owing to factors such as selection mechanisms, crossover strategies, population size, or coding errors. Furthermore, GAs are resource-intensive and require substantial computational power [10].

#### 3.1.3. K-Nearest Neighbors

K-Nearest Neighbor (k-NN) is a supervised learning algorithm used to classify an untagged substance by comparing its features to those of its closest neighbors. The algorithm relies on two key parameters (Figure 2b). The first is the number of neighbors, denoted by k, which determines the number of neighboring data points considered for comparison. The second parameter is the distance metric that measures the resemblance between the features of an object and its neighbors. To optimize the k-NN, a grid optimization was conducted to explore the values of k within the range of 2–21 [33].

#### 3.1.4. Decision Tree

A decision tree (DT) is a supervised learning algorithm that is applicable to both classification and regression tasks. It works by making a sequence of decisions that guide the model towards a particular outcome. In classification problems, decision trees divide the data into distinct groups based on the response variable and effectively sort them into different classes [54].

DT construction is a crucial process in machine learning that relies on training datasets (Figure 2c). It involves three primary steps: feature selection, DT generation, and pruning. Feature selection involves determining the most significant features in a dataset to efficiently divide the data, with each partition representing a leaf in the DT [65]. DT generation starts at the root node and recursively creates subnodes by classifying data based on selected features, continuing until the data are sufficiently partitioned [49,66]. To avoid overfitting, pruning is applied to simplify the tree by removing unnecessary branches, thus improving model generalization and reducing complexity [49].

As the number of parameters in microalgae biofuel studies increases, utilizing a decision tree structure becomes increasingly important for optimizing simulations under varying environmental conditions. Decision trees are highly efficient and capable of processing high-dimensional and large input datasets more quickly than many other methods [54]. However, a common issue with decision trees is their tendency to overfit, where the model is overly aligned with the training data, leading to diminished accuracy when predicting the outcomes for new data. Although this leads to a very low training error, the error in the test data can be significantly higher. To mitigate this problem, random forests can be employed, as they combine multiple decision trees through bootstrapping and aggregation and train several trees simultaneously to improve performance [54].

#### 3.1.5. Random Forest

The random forest (RF) algorithm combines elements of the Bagging technique and decision tree methodology, often using decision trees as the foundation for classification tasks [49]. Unlike traditional methods that train multiple decision trees on the same dataset, RFs select a random subset of features to construct each decision tree. This approach introduces variability among trees by choosing a different feature for splitting at each node within the trees (Figure 2d). As a result, RFs effectively reduce the likelihood of overfitting, particularly in the realm of ensemble learning [10,67].

RFs are particularly effective for handling larger datasets; however, creating a random forest model requires significant computational resources [11,57]. Compared to decision tree algorithms, RFs generally yield improved performance in both classification and regression tasks. In relation to other ML methods, such as SVMs and deep learning (DL) techniques, such as convolutional neural networks (CNNs), RFs typically offer faster prediction times and better accuracy while requiring less computational power [49]. Numerous studies have utilized both DT and RF algorithms to predict the outcomes of microalgae cultivation and extraction of bioproducts [10].

Although random forests are an ensemble method, they may not perform well on very complex tasks where dependencies within the data require deeper hierarchical feature extraction [68]. Advanced ensemble techniques, such as gradient boosting machines (GBM) and XGBoost, combine multiple models (often called “weak learners” or “base models”) to improve predictive performance [69]. Unlike methods such as random forest, these boosting algorithms build models sequentially. Each new model in the sequence focuses on correcting the errors made by previous models, allowing them to better capture complex relationships within the data. This process renders gradient boosting techniques more resistant to overfitting and enhances their ability to generalize well to new data. As a result, boosting methods often outperform random forests in tasks that require the precise handling of intricate patterns and dependencies in data [68,69].

### 3.2. Neural Networks

Neural networks, commonly known as artificial neural networks (ANNs), are black box algorithms commonly employed in machine learning. They rely on a gradient descent backpropagation process to adjust and optimize their performance [10,11]. These computational models aim to replicate the function of the human brain by connecting nodes or neurons in a complex web-like structure [54]. A network of interconnected neurons allows the system to learn and process information in a manner similar to that of biological neural systems. An ANN is a nonlinear, highly adaptive, fault-tolerant system [10,53]. To ensure that the model functions correctly, it is essential to provide high-quality and sufficient data, select an appropriate network structure, and perform proper training [70].

In an ANN, the operational conditions are considered inputs, while the analyzed results act as the outputs [45]. The ANN structure is composed of three key layers. The first layer, called the input layer, receives inputs and passes them to the hidden layer. The hidden layer is where the actual processing occurs, meaning that this is where predictions of the outputs based on the inputs are made (Figure 2e). The predicted outputs are then forwarded to the output layer. A comparison is made between the forecasted results and the observed results. This comparison evaluates the accuracy of the model, providing insight into the error, which is the difference between the forecasted and actual outcomes. Each of the three layers contains nodes, also referred to as neurons that facilitate the transfer of information from one layer to the next [45].

ANNs can be classified into two types: single-layer and multi-layer networks. In single-layer ANNs, an input neuron is linked directly to an output neuron. Conversely, multi-layer models include multiple hidden layers between the input and output layers [11]. The hidden layer is where the input processing occurs to predict the output using various transfer functions [45]. Transfer functions are crucial for enhancing prediction accuracy [71]. Common transfer functions include the hard limit function (hardlim), linear function (purelin), and logarithmic sigmoid function (logsig) [45].

The network architecture of an ANN is determined by how its layers are interconnected. This architecture includes the total number of layers in the network, number of neurons or nodes within each layer, transfer function used by each layer, and the connection of neurons within the layer. The strength of the interconnections between neurons holds the network information, allowing the ANN to be trained by adjusting these interconnection values, known as weights. The quantity of hidden neurons in the hidden layer is typically selected through a trial-and-error process. A model is considered to have strong predictive capability when the error approaches zero [45].

The development of an ANN model plays a crucial role in predicting experimental outcomes, not only for the experiments that were performed, but also for various untested working conditions. This capability is especially valuable during the process scale-up. At the pilot or industrial scale, processes tend to be highly dynamic, with constantly changing operating conditions. Implementing an ANN model allows for the ongoing optimization of these conditions, which leads to better control over product quality, helps mitigate process disturbances, and enables the process behavior to be accurately replicated [71].

ANNs excel at handling and modeling intricate interactions within systems. One key advantage is their flexibility, which allows them to adjust to new information as it evolves over time [57]. This adaptability makes ANNs particularly effective for studying microalgae processes, where interactions are often highly complex [57]. However, the use of a high number of neurons in the hidden layer can result in time-intensive training and an increased likelihood of issues such as overfitting or getting stuck in the local minima. Therefore, selecting the right number of neurons and hidden layers is crucial for effective ANN modeling [72,73].

A notable drawback of neural networks is their inability to supply transparent explanations in decision making. They rely solely on empirical data, which means that they do not offer insight into the underlying mechanisms that drive changes. This limitation makes them unsuitable for gaining a mechanistic understanding of processes [10,53]. Moreover, the intricacy of ANN architectures introduces further challenges, as it requires optimizing various factors like data partitioning, pre-processing, hyperparameter optimization, and model evaluation [30]. To overcome these limitations, a deep learning algorithm is employed as an alternative. This approach helps reduce issues related to the vanishing gradient effect [45].

#### Adaptive Neuro-Fuzzy Inference System

It is important to mention at this point that the Adaptive Neuro-Fuzzy Inference System (ANFIS) is a hybrid algorithm. The ANFIS integrates the rapid capabilities of neural networks with the principles of fuzzy inference systems. Utilizing these soft computing approaches allows the creation of a black box model without relying on mathematical formulations [74,75]. While a fuzzy system on its own struggles to yield accurate results owing to its inability to adjust membership functions automatically, the ANFIS overcomes this limitation. Additionally, the ANFIS offers superior smoothness compared to ANNs and is well equipped to tackle complex engineering challenges [23].

### 3.3. Deep Learning

Deep learning (DL) involves the use of neural networks with multiple layers [41,76]. The goal of DL is to discover hidden patterns in data by first identifying specific low-level features and then progressively combining them into more abstract higher-level representations [77]. One major benefit of DL is that its performance tends to improve as more data become available. However, for deep learning to be effective, it requires large datasets, often consisting of thousands of images, as well as a graphical processing unit (GPU) to efficiently handle the data and train the model [78]. Ensemble approaches, when combined with DL algorithms, provide a robust framework that improves model accuracy, generalization, and stability, often outperforming traditional individual models, such as like SVMs, ANNs, and GAs, on complex datasets [68,69,79].

DL algorithms and methods can be applied to various tasks related to microalgae, including classification, identification, and segmentation. DL can be employed in different learning paradigms such as supervised, unsupervised, and hybrid learning approaches [78]. The most common types of DL are convolutional neural networks (CNNs), recurrent neural networks (RNNs), and autoencoders (AE) [10].

#### 3.3.1. Convolutional Neural Networks

Convolutional neural networks (CNNs) consist of a series of convolutional layers, each with a local receptive field, combined with pooling layers that perform down-sampling. Although CNNs are primarily used in image recognition tasks, they are also effective in video interpretation and language processing [80]. The convolutional layers are responsible for detecting the features, extracting essential information from the input data, and introducing nonlinearity into the features through an activation function [49].

#### 3.3.2. Recurrent Neural Networks

Recurrent neural networks (RNNs) convert input data into a format that can be processed by the network and determine how to integrate the data recursively through recurrent units. They employ a loss function to quantify the difference between the forecasted and actual values and use the backpropagation algorithm to calculate the gradient, enabling the network parameters to be updated. Once training is finished, the model’s performance is assessed using validation and test datasets to detect overfitting or underfitting and to implement required adjustments [43].

#### 3.3.3. Autoencoders

The initial component of the autoencoder (AE), known as the encoder, converts the input data into a condensed intermediate representation by reducing the data dimensions and identifying the essential features. Following this step, the data passes through a constricted layer called the bottleneck layer, which compresses the information further and prompts the network to concentrate on the most important features. In the third part, the decoder receives the encoded data and rebuilds the original input. During the training, the AE is optimized to ensure the output closely matches the original input. The backpropagation algorithm is used to calculate the gradient, whereas an optimization algorithm adjusts the network weights to minimize the reconstruction error [43].

## 4. Intersection of IoT and AI/ML

The IoT and ML are distinct technological applications. While the IoT enables interconnectivity between various devices through the Internet, ML applies artificial intelligence to introduce intelligence into microalgae operations. The IoT operates by installing sensors that collect data, which are stored in cloud databases and can be accessed through devices such as smartphones or laptops [8]. The vast datasets produced by these monitoring systems serve as inputs for MLs [10]. ML then optimizes these data, supporting production efficiency, and enabling accurate production forecasts [8]. These technologies reduce manual effort and improve the system performance in microalgae processing [44].

The incorporation of IoT and AI-driven technologies into the plants, has revolutionized industrial processes by improving design flexibility, lowering production costs, and accelerating production times [81]. In the microalgae industry, IoT-based systems help simplify and optimize production, leading to more sustainable and efficient practices [8,82]. Furthermore, AI and ML in smart control systems minimize resource use and aid microalgae biorefineries in making informed decisions [11]. These advancements have significant opportunities for enhancing the productivity and sustainability of microalgae-based industries.

Although the installation of hardware components necessary for the IoT may raise concerns about maintenance costs, studies indicate that the IoT and AI can significantly reduce these expenses [8]. Specifically, IoT and AI implementation in factories has been reported to lower maintenance costs by 12–40%, minimize equipment downtime by 50%, and extend the lifespan of machinery by 20%. Additionally, these technologies can reduce risks related to safety, health, the environment, and quality by 14% and decrease capital investments in equipment by 3% to 5% [83]. Thus, the integration of the IoT and AI enhances operational efficiency and provides sustained economic advantages [8].

However, the integration of IoT devices in industrial processes raises security concerns, particularly regarding data privacy. IoT devices, which are connected to various sensors for monitoring production systems, can be accessed by manufacturers or designers of these devices via the Internet. This problem poses potential risks to data security. In addition, IoT-enabled systems face challenges related to data management and timely technical support. Another obstacle is the dependency on Internet connectivity, making it difficult to implement IoT applications in remote or underdeveloped regions that lack reliable Internet access. Despite these challenges, the use of the IoT remains promising for advancing industrial processes, provided that the security and connectivity issues are adequately addressed [81].

Recently, numerous researchers have investigated the use of smart systems in microalgae production. The use of sensors can greatly improve productivity [10]. ML models can also be developed from collected data to optimize performance. One study described the application of AI-based sensors to monitor and control a co-culture system, gathering data on factors such as pH, nutrient concentrations, and dissolved oxygen. This information was fed into an AI model that used deep learning techniques to predict optimal conditions for the process, ultimately enhancing both performance and energy efficiency [44,84]. Tham et al. [85] created an IoT-enabled pilot-scale PBR that allows for the remote monitoring of cultivation factors through a smartphone. It was found that integrating the IoT into microalgae processes reduced production input by 30% and increased output by 20% [86]. Additionally, Giannino et al. [87] demonstrated that systems utilizing the IoT and AI-based monitoring showed a 9% increase in production compared to those without such monitoring [8].

## 5. Applications of AI/ML in Microalgae Processes

### 5.1. Classification

Microalgae are categorized based on distinct traits such as size, texture, color, and shape, which can typically be observed using an optical microscope. However, traditional identification methods tend to be expensive, time-consuming, error-prone, and require skilled taxonomists [88]. DNA-based identification is favored for these strains that lack distinct microstructural features, show minimal phenotypic differences at the species and genus level, and exhibit changes in shape characteristics [58]. Despite their advantages, DNA-based methods involve complex procedures and laborious optimization steps, require specialized equipment, and are often time-consuming and costly to validate [58,89].

However, advances in ML, which have significantly improved fields such as digital image processing and speech recognition [90], have the capability to simplify and automate these complex processes [49]. The application of AI and ML in microalgae classification aims to integrate AI technologies with an understanding of the shape, texture, and convolutional features of microalgae. This approach can significantly accelerate real-time monitoring and enable the rapid and accurate identification of species [58]. Several researchers have introduced ML techniques to the microalgae field to classify species [49,91]. The study utilized microalgae features, including diameter, aspect ratio, width, and length, as input variables for ML models [11].

The neural network model, trained on a large dataset from the FlowCam device (Yokogawa Fluid Imaging Technologies, Inc., Maine, USA), showed enhanced reliability and performance in classifying various genera, including *Chlorella*, *Scenedesmus*, *Haematococcus*, *Synechococcus*, *Chlamydopodium*, and *Docystidium*, thereby improving its classification accuracy. For each species, three samples were introduced into FlowCam, generating 150,000 images per species. Of these, 15% (22,500 images per class) were used, and the remaining 15% allocated to the test set. The training process spanned six epochs or iterations over the entire dataset. The model achieved a final accuracy of 83.43% without classification thresholds, which increased to 97.27% when the thresholds were applied [70]. As reported by Chong et al. [58], *Chlorella vulgaris* FSP-E, *Chlamydomonas reinhardtii*, and *Spirulina platensis* were selected for the classification of dead and living microalgae. The results showed that the final set of combined features, along with optimized image pre-processing techniques, achieved high accuracy rates of 96.93% with the k-NN classifier and 97.63% with the SVM classifier.

Giraldo-Zuluaga et al. [92] implemented the ANN and SVM as pattern recognition methods to create a system for the automated detection of *Scenedesmus coenobia* through a microscopic image analysis. The approach achieved accuracy rates of 98.63% with the SVM and 97.32% with the ANN [10]. In another study, the AlexNet-SVM model was utilized to classify the images of microalgae from the Cyanobacteria and Chlorophyta groups [93]. In this study, 472 microalgal images were used, with 203 from the Cyanobacteria group and 269 from the Chlorophyta group. The deep features extracted from these images were inputted into the SVM for classification. Several kernel functions, including Gaussian, Cubic, Quadratic, and Linear functions, were applied to train the SVM classifier using deep features. The highest classification accuracy of 99.66% was achieved using the cubic kernel function. By incorporating deep features from a model with an initially lower accuracy, the use of the SVM significantly improved the overall performance [93].

Zheng et al. [94] introduced an Automated Intelligent Microfluidic Platform (AIMP) designed to identify and classify four different types of microalgae: *Cosmarium*, *Closterium*, *Micrasterias*, and *Haematococcus pluvialis*. This system employs automated control and advanced data analysis to perform its functions. Researchers began with 812 captured images, which resulted in the extraction of 630, 770, 737, and 736 labels for *Cosmarium*, *Closterium*, *Haematococcus pluvialis*, and *Micrasterias*, respectively, thus forming the raw image dataset. Utilizing this dataset, the microalgae species detection network (MSDN), which is based on the YOLOv5 architecture, achieved an impressive accuracy of 92.8%. Although the current functionality of the instrument is constrained by a relatively limited dataset, improvements are anticipated as more high-quality data are incorporated [43].

### 5.2. Upstream Microalgae Processes

The cultivation stage plays a crucial role in microalgae biomass production [8]. Numerous researchers have utilized ML models to enhance the process by forecasting the ideal combination of growth factors. Factors such as the type of bioreactor, temperature, pH, inoculum, light availability, nutrient content, and CO_2_ levels are highly dependent on the microalgae species used. Hence, providing optimal conditions is critical for maximizing treatment efficiency and enhancing biomass production [11].

For example, a feedforward backpropagation ANN was used to forecast the dry cell weight of microalgae using six input variables: temperature, pH, dissolved oxygen (DO), electrical conductivity (EC), NO^3−^, and PO_4_^3−^. The network was trained using 35 experimental results, and the dataset was divided into 70% for training, 15% for cross-validation, and 15% for testing. The optimized ANN architecture (six input nodes, ten hidden nodes, and one output node), trained using the Levenberg–Marquardt algorithm, demonstrated excellent predictive accuracy, achieving an *R*^2^ value of 0.983 [95].

As reported by Onay [96], the RSM and ANN were employed to predict the maximum lipid content in *Chlorella minutissima* (Table 2). The neural network was trained using the Levenberg–Marquardt algorithm. The model consisted of an input layer, a hidden layer, and an output layer. The input layer had three neurons, representing wastewater concentration, chitinase, and lysozyme, whereas the hidden layer comprised 20 neurons. The output layer had a single neuron. The results showed that the ANN model, with an *R*^2^ value of 0.9634, provided better predictions of lipid content than the RSM model.

Another study evaluated the performance of four ML algorithms, ANN, CNN-1D, k-NN, and RF, in modeling carbohydrate buildup in a mixed cyanobacterial community grown in domestic wastewater. The models were designed to account for interactions between carbon, substrate, growth, and population dynamics. Among the algorithms tested, the CNN-1D model exhibited the greatest forecasting accuracy, with an *R*^2^ value of 0.8966, effectively approximating the behavior of the system [33]. These findings highlight the potential of CNN-1D to accurately model carbohydrate accumulation in complex biological systems.

In a study, the CO_2_ fixation rates of various algal strains were gathered. Production conditions, such as pH, CO_2_ concentration, temperature, and nitrogen and phosphorus levels (in mg/L) were used as input variables. The CO_2_ fixation rate was considered the output variable. A dataset of 61 data points compiled from diverse literature sources was used to assess the accuracy of both the ANFIS and GA-ANFIS models. The GA-ANFIS model, with an *R*^2^ value of 0.9846, demonstrated superior predictive performance compared with the standard ANFIS model [23]. On the other hand, Hossain et al. [99] examined the effects of light–dark cycles, temperature, and nitrogen–phosphorus ratios on the CO_2_ biofixation of *Chlorella vulgaris* microalgae. ANN, boosted regression tree (BRT), and support vector regression (SVR: an extension of SVM) models were employed, and each AI method was enhanced using the Bayesian optimization algorithm (BOA) to predict CO_2_ biofixation. The SVR model achieved a high *R*^2^ value of 0.911. Additionally, the fractional bias was close to zero (0.0088), indicating the reliability of the model.

Saini et al. [100] reported that a hybrid algorithm CNN-GA was applied to optimize input parameters to maximize phycobiliprotein (PBP) production and cell growth in *Nostoc* sp. CCC-403. The model focused on three BG-11 media components (FAC, K_2_HPO_4_, and MgSO_4_) and the pH as input factors. The CNN-GA predicted the optimal conditions, resulting in a 90% increase in biomass yield and 61.76% enhancement in PBP recovery. This study demonstrated the effectiveness of the CNN-GA approach in optimizing cultivation parameters for improved biological production.

### 5.3. Downstream Microalgae Processes

**Figure 3:**
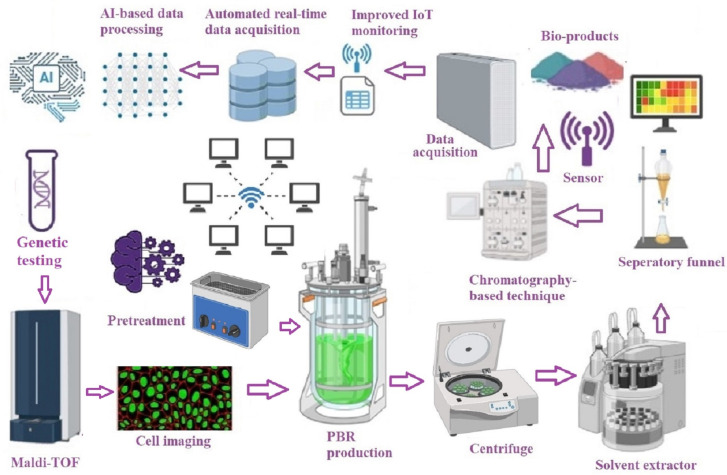
AI/ML applications in microalgae processes (Created with Biorender).

The downstream processing of microalgae, which includes the harvesting, extraction, and purification of valuable biomolecules, is a critical step in the production of biofuels, pharmaceuticals, and nutraceuticals (Figure 3). However, the optimization of these processes is complex because of the variability in microalgae species, separation/extraction conditions, and product yields. AI/ML models have become valuable tools for addressing bottlenecks in bioactive compound extraction and quantification by introducing innovations that incorporate digitalization, large-scale data, and automation to enhance effectiveness [30]. AI/ML models can be particularly effective in improving microalgal harvesting and significantly reducing the associated costs [11]. Additionally, there is an increasing demand to lower input costs in the extraction process [30,32]. AI/ML techniques such as the ANFIS, ANN, and SVM are widely used for the identification, measurement, and forecasting of bioactive compounds because of their advantages, including reduced duration, lower toxic solvent use, sufficient accuracy, strong predictive capabilities, cost-effectiveness, and more sustainable solutions [30].

In a study, AI models were utilized to analyze and enhance the efficiency and effectiveness of the vacuum drying process for Chlorococcum infusionum. ANNs and SVMs were applied to improve process efficiency. The input variables included temperature and pressure at time t, along with delta moisture (%) at time t − 1, while the output variable was delta moisture (%) at time t. The ANN showed superior performance, achieving significantly lower error values than the SVM [101]. In another study, Sultana et al. [102] applied ANNs and SVRs to predict biodiesel production from Nannochloropsis oculate. The input parameters included the catalyst dosage, reaction time, reaction temperature, and oil-to-methanol ratio, while the output was the biodiesel yield. To optimize the model, the SVR hyperparameters were automatically fine-tuned using a Bayesian algorithm. The SVR model exhibited better performance, with an *R*^2^ value of 0.991.

As reported by Sarkar et al. [103], the ANN was used to model the extraction yield of chlorophyll and carotenoids from Chlorella thermophila. The optimization focused on extraction factors, such as the homogenization duration, homogenization rate, microwave duration, temperature, solid-to-solvent ratio, and boiling time. A feedforward backpropagation network with one hidden layer, trained using the Levenberg–Marquardt algorithm, was employed for accurate predictions. In this study, 70% of the 138 data points were randomly chosen for model training, whereas 15% were used for cross-validation to avoid overfitting. The ANN model, which included six input parameters and nine neurons in the hidden layer, was created to predict two outputs, chlorophyll and carotenoid yields, and achieved a high correlation coefficient (*R*^2^ = 0.98302).

The integration of an ANN for data processing and analysis with a GA for parameter optimization can enhance the prediction and optimization processes [30,104]. Srivastava et al. [39] focused on enhancing the conversion of microalgae oil into fatty acid methyl esters using supercritical methanol transesterification. It employs a sequential hybrid approach integrating the RSM, ANN, and GA to maximize conversion efficiency. The key process parameters (temperature, duration, and methanol/oil molar ratio) were optimized through a sequential process. Preliminary experiments defined the boundary conditions, and the RSM provided a precise experimental matrix. An ANOVA validated the accuracy of the model, whereas the ANN addressed nonlinear interactions. The optimized conditions predicted by the ANN were used as a fitness function for the GA to identify the global optima. A three-layer neural network was employed in the ANN, utilizing a log-sigmoid (logsig) transfer function in the hidden layer and a linear (purelin) transfer function in the output layer. The sequential hybrid RSM-ANN-GA approach achieved a predicted conversion efficiency of 99.16%, with experimental validation yielding a conversion efficiency of 98.12%. This integrative optimization methodology improves the understanding of transesterification parameters and promotes sustainable process development.

Mayol et al. [105] integrated AI with life cycle analysis to evaluate the environmental effects of converting biomass into biofuels, specifically biodiesel. The ANFIS was used to forecast environmental effects at various processing stages. The ANFIS model utilized seven environmental inputs, the power used in cultivation and transesterification, methanol and heat in transesterification, solid and liquid residue inputs in biochar and anaerobic digestion, and methane in combined heat and power, to predict the environmental index, specifically the global warming potential. The results revealed how each input parameter influenced the environmental impact of the system [11]. This approach offers valuable insights for optimizing biofuel production processes while minimizing their environmental footprints.

Recent studies have highlighted that AI-driven advancements in microalgae manufacturing, both in upstream and downstream processing, are progressing swiftly, propelled by innovations in machine learning, biotechnology, and automation [106,107]. AI-driven predictive maintenance systems help manufacturers predict and prevent equipment breakdowns in advance, significantly reducing downtime and costs. Algorithms analyze sensor data in real time to forecast the likelihood of machine malfunctions, which enables a proactive rather than a reactive approach to equipment management [108]. AI/ML algorithms make supply chains more adaptive and resilient by predicting demand and optimizing logistics [109]. The use of digital twins, virtual replicas of physical systems, is a breakthrough in manufacturing. AI-powered digital twins allow manufacturers to simulate, analyze, and optimize processes before implementing them in the real world, thereby speeding up innovation and reducing costs [110]. In manufacturing, AI/ML techniques reduce production costs and waste, while improving product quality and supply chain resilience [109]. These advancements underscore the importance of continued research, development, and cross-disciplinary collaboration to harness the potential of AI in these sectors fully.

## 6. Ethical Issues and Challenges

The application of AI/ML in microalgae processes raises several ethical concerns, such as the transparency and accountability of AI decision-making processes and discriminatory outcomes in environmental protection measures. For instance, if a model is trained on data that exclusively cover microalgae species from certain geographic regions, it might not generalize well when applied to different environments. Furthermore, data privacy and ownership are important factors, especially when combined with the IoT for real-time data collection. Job displacement is another ethical problem. It is also crucial to determine who is responsible for the errors or unintended consequences in AI/ML applications.

In industries in which decisions impact safety or human well-being, the interpretability of AI/ML models is critical [111,112]. Black box models may not provide sufficient insight into why decisions are made, which can lead to ethical concerns [113]. Ensuring that AI/ML systems do not introduce or amplify bias is vital because biased outcomes can have serious social implications [114]. Additionally, in industrial environments where AI/ML systems control or influence physical processes, such as in manufacturing or autonomous vehicles, safety becomes paramount. Errors in AI/ML predictions or decisions can lead to accidents or equipment damage, potentially endangering their lives. It is crucial to maintain safety demands through the testing, validation, and ongoing monitoring of AI/ML systems to detect and address failures before they can cause harm. Industries must adopt robust safety standards and fail-safe mechanisms to minimize the risk of accidents and uphold their duties to protect employees, customers, and the general public [111,112]. By incorporating these ethical principles, industries can foster trust, enhance safety, and contribute positively to society, while maximizing the benefits of AI/ML technologies.

The other issue is the protection of trade-off between energy use and environmental benefits. For example, the high energy consumption required for running advanced AI models could counteract some of the environmental benefits derived from optimizing microalgae production, especially if the energy source is not renewable. Optimizing computational processes is an effective way to reduce environmental impact. In addition, AI/ML can support sustainability efforts by improving efficiency, reducing waste, and conserving resources. However, it is crucial to verify that the environmental advantages gained from implementing these technologies are greater than their associated costs [115].

Addressing these ethical issues requires balancing innovation and responsibility. As AI/ML continues to be integrated into microalgae processes, stakeholders, including researchers, companies, and governments, must ensure that these technologies are applied in ways that are sustainable, fair, and transparent.

The main challenges for the application of AI/ML in microalgae processes are summarized as follows:

Data Scarcity: Machine learning models require large datasets for effective training; nevertheless, obtaining such data can be both time-intensive and expensive. Techniques, such as data augmentation and pre-processing, help alleviate this issue [10].

Data Quality and Noise: Even when data are available, they may contain errors, inconsistencies, or noise, which can degrade the model performance. Ensuring high-quality clean data is critical for accurate prediction [11].

Model Complexity: Some AI/ML models demand significant computational power, making them difficult to implement, particularly in resource-limited environments [44].

Cost of Implementation: Beyond computational resources, implementing AI/ML systems often involves additional costs, such as sensor networks, cloud storage, and maintenance. These additional costs can be prohibitive for smaller companies or research institutions [44].

Lack of Standardization: The absence of standardized methods for applying AI/ML models, particularly in niche areas such as microalgae processes, hampers result comparisons and practical adoption [44].

Scalability and Efficiency: Creating large-scale AI models for real-world industrial implementations requires careful consideration of the resources, scalability, and efficiency [30].

Choosing the Right Algorithm: Selecting an suitable ML algorithm for particular tasks can be challenging, especially in emerging areas such as microalgae pigment extraction [11].

IoT Connectivity and Cybersecurity: Lack of Internet access in rural areas hinders IoT implementation, and cybersecurity risks pose additional concerns for companies relying on cloud-connected systems [8].

Skill Shortage: A lack of professionals skilled in IoT and AI fields affects industries such as microalgae, necessitating more funding and training opportunities [8].

Industrial Data Management via AI/ML: Industrial AI/ML systems often require large volumes of high-quality data; however, collecting, cleaning, and curating data can be difficult and costly. Many industrial settings lack centralized data repositories or have data in incompatible formats, which complicates the integration. Industrial environments are often subject to changes over time, such as machinery wear and tears, seasonal variations, and changing market demand. AI/ML models can degrade in performance if they are not continually updated or retrained to adapt to evolving conditions. In industries that handle sensitive information, managing data security and complying with privacy regulations can be significant obstacles [116,117].

In addition to the challenges outlined by the above authors, a few other issues could affect AI/ML applications in microalgae processes, as follows:

Biological variability: Microalgae processes can be highly variable due to biological factors such as growth conditions, genetic differences, and environmental changes. This variability adds complexity to model development and prediction accuracy.

Regulatory Issues: The use of AI/ML in microalgae processes might face regulatory challenges, especially concerning the use of automated decision making in critical processes, such as food or biofuel production.

These factors collectively shape the landscape of AI/ML applications in microalgae processes. To overcome these challenges, simulation-based data generation can be applied to create synthetic datasets, and model compression or distillation techniques can be used to simplify models without significantly sacrificing their performance.AI/ML systems with modular components can be designed to be independently scaled, allowing incremental upgrades and efficiency improvements. Alternative connectivity options, such as satellite Internet or mesh networks, can be explored to ensure reliable IoT deployment in rural areas. Adaptive learning systems that can dynamically adjust models based on real-time data can be utilized to account for biological variability. By addressing these recommendations, stakeholders can better address the challenges related to AI/ML applications in microalgae processes, ultimately enhancing productivity and innovation in the field.

## 7. Conclusions and Outlook for the Future

The integration of AI/ML technologies in microalgae processes is an emerging field with significant potential. This review offers a new viewpoint on how data-driven methods can enhance the efficiency of microalgae processes. The key findings from this review are outlined below in bullet points.

- AI/ML technologies in microalgae processes offer data-driven optimization, surpassing traditional methods in terms of efficiency, yield, and control.
- Key applications include species identification, the optimization of growth conditions, harvesting, extraction, and purification in microalgae processes.
- Popular ML algorithms used are SVM, GA, DT, RF, ANN, and DL, each with their strengths and limitations.
- AI/ML enhances performance, stability, and scalability and reduces manual labor, costs, downtime, and environmental risks.
- The challenges include data limitations, model complexity, scalability, cybersecurity, and regulatory concerns.
- Solutions, such as simulation-based data, modular design, and adaptive learning models, can overcome these challenges and foster innovation.

Future studies should investigate the use of hybrid AI models that combine supervised and unsupervised learning techniques to identify genetic markers associated with high lipid production in microalgae. IoT sensors can gather data throughout the lifecycle of microalgae cultivation, from growth to harvesting and processing. Drone-based remote sensing technologies combined with AI/ML algorithms can be implemented to observe large-scale microalgae farms. AI/ML algorithms can be employed to assess market trends, consumer preferences, and social media data to forecast demand for microalgae-derived products (e.g., health supplements and biofuels). These examples illustrate how AI/ML and the IoT can be synergistically applied to advance microalgae processes, ultimately leading to enhanced productivity, sustainability, and innovation in this field.

## Figures and Tables

**Table 1:** Merits and demerits of AI/ML algorithms.

AI/ML Algorithms	Merits	Demerits
Support Vector Machine	Flexible Capable of managing high-dimensional data Excellent precision Well-suited for tasks involving binary classification	Sensitive to nonlinear kernel functions Requiring greater computational resources Time consuming in processing large datasets Low training efficiency
Genetic Algorithm	Avoiding local minima Versatile Optimize problems involving multiple variables No need for data pre-processing	Risk of premature convergence Requiring greater computational resources Time consuming
K-Nearest Neighbor	Simple implementation Well-suited for multi-layered data	Needs distance computation
Decision Tree	Simpler for handling quantitative and specific data Data scaling is not required Missing data can be handled	Requiring larger dataset Risk of overfitting Challenging to control the size of the tree
Random Forest	Highly flexible Resistant to overfitting Quicker to train Efficient for nonlinear data	Not ideal for small-sized data variables Computationally intensive Inadequate convergence
Artificial Neural Network	Highly adaptive Fault-tolerant system Skilled at capturing complex, multilayered interactions Helps mitigate process disturbances	Risk of overfitting Requiring data pre-processing Time-consuming training Complexity of ANN architectures

**Table 2:** The role of AI/ML in microalgae processes.

Process	AI/ML Algorithms	Application	Strain	Accuracy	Reference
**Classification**	RF	Classifying dead or alive microalgae populations	*Chlorella vulgaris*	94.50%	[97]
	CNN	Classification	*Acutodesmus obliquus*, *Monoraphidium* sp., *Spirullina* sp., *Tetradesmus deserticola*, *Desmodesmus perforatus*	89.00%	[98]
	ANN	Classification	*Chlorella*, *Scenedesmus*, *Haematococcus*, *Synechococcus*, *Chlamydopodium*, and *Docystidium*	97.27%	[70]
	SVM	Classification	Cyanobacteria and Chlorophyta	99.66%	[93]
	k-NN	Classification	*Chlorella vulgaris* FSP-E, *Chlamydomonas reinhardtii*, and *Spirulina platensis*	96.93%	[58]
**Upstream** **microalgae processes**	ANN	Optimization of wastewater concentration, chitinase, and lysozyme for lipid content	*Chlorella minutissima*	96.34%	[96]
	ANN	Optimization of temperature, pH, DO, EC, NO^3−^, and PO_4_^3−^ to predict dry cell weight	*Scenedesmus* sp., and *Chlorella* sp.	98.30%	[95]
	SVR	Examination of the effects of temperature, light–dark cycles, and nitrogen–phosphorus ratios on the CO_2_ biofixation	*Chlorella vulgaris*	91.10%	[99]
	GA-ANFIS	Evaluation of temperature, pH, CO_2_, and nitrogen and phosphorus levels to predict CO_2_ fixation rates	Various algal strains	98.46%	[23]
	CNN-GA	Optimization of BG-11 media components and pH to maximize PBP production and cell growth	*Nostoc* sp. CCC-403	-	[100]
**Downstream** **microalgae processes**	ANN	Evaluation of temperature, pressure, and moisture content to predict the efficiency of the vacuum drying process	*Chlorococcum infusionum*	-	[101]
	SVR	Examination of the catalyst dosage, reaction time, reaction temperature, and oil-to-methanol ratio to predict biodiesel yields	*Nannochloropsis oculate*	99.10%	[102]
	ANN	Optimization of extraction parameters to predict the yields of chlorophylls and carotenoids	*Chlorella thermophila*	98.30%	[103]
	RSM-ANN-GA	Optimization of temperature, time, and methanol/oil molar ratio to predict conversion yield in transesterification	*Chlorella* CG12	99.16%	[39]

## Data Availability

Not applicable.
